# Multicentred randomised controlled trial of an augmented exercise referral scheme using web-based behavioural support in individuals with metabolic, musculoskeletal and mental health conditions: protocol for the e-coachER trial

**DOI:** 10.1136/bmjopen-2018-022382

**Published:** 2018-09-21

**Authors:** Wendy Ingram, Douglas Webb, Rod S Taylor, Nana Anokye, Lucy Yardley, Kate Jolly, Nanette Mutrie, John L Campbell, Sarah Gerard Dean, Colin Greaves, Mary Steele, Jeffrey D Lambert, Chloe McAdam, Ben Jane, Jennie King, Ray B Jones, Paul Little, Anthony Woolf, Jo Erwin, Nigel Charles, Rohini H Terry, Adrian H Taylor

**Affiliations:** 1 Faculty of Medicine and Dentistry, Peninsula Medical School, University of Plymouth, Plymouth, UK; 2 University of Exeter Medical School, Exeter, UK; 3 Department of Clinical Sciences, Brunel University, London, UK; 4 Faculty of Medicine, Southampton University, Southampton, UK; 5 Nuffield Department of Primary Care Health Sciences, Oxford University, Oxford, UK; 6 School of Sport, Exercise and Rehabilitation Sciences, University of Birmingham, Birmingham, UK; 7 Physical Activity for Health Research Centre, University of Edinburgh, Edinburgh, UK; 8 Faculty of Sport and Health, University of St Mark and St John, Plymouth, UK; 9 Patient & Public Involvement, Plymouth, UK; 10 Department of Rheumatology, Royal Cornwall Hospitals NHS Trust, Truro, UK

**Keywords:** primary care, hypertension, general diabetes, mental health, musculoskeletal disorders

## Abstract

**Introduction:**

Physical activity is recommended for improving health among people with common chronic conditions such as obesity, diabetes, hypertension, osteoarthritis and low mood. One approach to promote physical activity is via primary care exercise referral schemes (ERS). However, there is limited support for the effectiveness of ERS for increasing long-term physical activity and additional interventions are needed to help patients overcome barriers to ERS uptake and adherence.

This study aims to determine whether augmenting usual ERS with web-based behavioural support, based on the LifeGuide platform, will increase long-term physical activity for patients with chronic physical and mental health conditions, and is cost-effective.

**Methods and analysis:**

A multicentre parallel two-group randomised controlled trial with 1:1 individual allocation to usual ERS alone (control) or usual ERS plus web-based behavioural support (intervention) with parallel economic and mixed methods process evaluations. Participants are low active adults with obesity, diabetes, hypertension, osteoarthritis or a history of depression, referred to an ERS from primary care in the UK.

The primary outcome measure is the number of minutes of moderate-to-vigorous physical activity (MVPA) in ≥10 min bouts measured by accelerometer over 1 week at 12 months.

We plan to recruit 413 participants, with 88% power at a two-sided alpha of 5%, assuming 20% attrition, to demonstrate a between-group difference of 36–39 min of MVPA per week at 12 months. An improvement of this magnitude represents an important change in physical activity, particularly for inactive participants with chronic conditions.

**Ethics and dissemination:**

Approved by North West Preston NHS Research Ethics Committee (15/NW/0347). Dissemination will include publication of findings for the stated outcomes, parallel process evaluation and economic evaluation in peer-reviewed journals.

Results will be disseminated to ERS services, primary healthcare providers and trial participants.

**Trial registration number:**

ISRCTN15644451; Pre-results.

**Strengths and limitations of this study**

- This is the first study to determine whether adding web-based interventions to primary care exercise referral schemes increases objectively assessed physical activity more than usual exercise referral schemes, after 1 year.
- The study includes inactive adults with one or more common chronic conditions.
- No physical health measures (except self-reported weight) were assessed in the study.
- It is expected that participants will have multiple chronic conditions, meaning the study may not be able to determine intervention effects on physical activity for each condition.
- Participants in the intervention arm will be invited to take part in in-depth qualitative interviews which may act as a cointervention.

## Introduction

Physical inactivity was found to cost the National Health Service (NHS) £455 million in 2013–2014 according to data collected by Clinical Commissioning Groups in the UK.^1^ Evidence-based guidelines recommend both aerobic and strength training for improving health markers and quality of life among those with common chronic metabolic conditions^2–5^ and musculoskeletal conditions,^6^ and mostly aerobic exercise for preventing and reducing depression.^7^ Public health guidelines of 150 min of moderate-to-vigorous physical activity (MVPA) per week are widely accepted but even small increases in physical activity and reduced sedentary time among the least active are likely to accrue health benefits.^8 9^

Patients with obesity, hypertension, type 2 diabetes, osteoarthritis and depression are less physically active than the general population,^2^ and need greater support to overcome real and perceived barriers to increase physical activity. Increases in physical activity among the least active have the potential to provide the largest impact on health but any benefits dissipate without maintained levels of activity.^10^ A variety of initiatives have been explored to promote physical activity within primary care, including referring patients to ‘exercise on prescription’, that is, an exercise referral scheme (ERS). In the UK, ERS have been common for promoting physical activity, with an estimated 600 schemes involving up to 100 000 patients per year.^11^

Evidence from a meta-analysis of eight randomised trials involving 5190 participants eligible for ERS^12^ indicated a small increase in the proportion of participants who achieved 90–150 min of physical activity of at least moderate intensity per week, compared with no exercise control at 6–12 months follow-up among at-risk individuals. But uncertainty remains regarding the effects for patients with specific medical conditions since no study assessed long-term physical activity objectively, and many of the eight studies reviewed had relatively small sample sizes.

A systematic review^13^ reported an average ERS uptake (attendance at the first ERS session) that ranged from 66% in observational studies to 81% in randomised controlled trials, and average levels of adherence from 49% in observational studies to 43% in randomised controlled trials. Predictors of uptake and adherence have rarely been explored but it has been reported that while women were more likely to begin an ERS, they were less likely to adhere to it than men; also, older people were more likely to begin and adhere to an ERS.^13^ ERS may help patients become familiar with concepts such as exercise type, intensity, frequency and duration of exercise, matched to their medical condition, and target key processes of behaviour change. However, the following features of an ERS may reduce uptake and adherence: inconvenience, cost, limited sustainable physical activity support (eg, for 10 weeks) and low appeal for structured exercise and/or the medical model, that is, ‘exercise on prescription’, which may do little to provide autonomous support nor empower patients to develop self-determined behaviour to manage chronic medical conditions.^11 14^ It therefore appears that additional support may be needed which is accessible, low cost, can be tailored to support a wide range of individual needs and empowers patients to develop and use self-regulatory skills (eg, self-monitoring, goal setting) to self-manage their chronic conditions. A wide variety of online and mobile technologies have been developed and used to support changes in and maintenance of physical activity.

There is considerable evidence on the effects of technology-based interventions for promotion of physical activity.^15 16^ These include studies with a wide range of interventions (from quite simple self-monitoring to interventions with complex multiple behaviour change components), targeted at different clinical groups with different baseline levels of physical activity, with various physical activity outcomes reported (very few using objective measures), and with mostly short-term follow-ups. Also, some comparisons are between intervention versus no intervention and others versus human contact, although none reports on the effects of adding web-based support to ERS. The impact for web-based and technology interventions on increasing physical activity is small to moderate (an effect size ≤0.4). However, there is evidence that more rigorous studies, interventions with more behaviour change components and ones targeted at less active populations are more effective.^15 16^ A systematic review^17^ has highlighted the importance of maximising sustained engagement in web-based interventions for enhancing change in the target behaviour. A recent study^18^ confirmed that self-monitoring of physical activity and tailored feedback were important to increase engagement, and periodic communications helped to maintain participant engagement.

The LifeGuide platform (www.LifeGuideonline.org/) has been extensively used to develop and evaluate acceptability and impact of online behaviour change and self-management interventions with a variety of clinical groups, including in primary care.^19–21^ For example, adding online LifeGuide support to face-to-face support showed a greater lasting reduction in obesity than face-to-face dietetic advice alone.^22^ The LifeGuide platform provides a researcher-led tool to develop interventions drawn from theory and evidence of effective techniques^23 24^ and provides the opportunity to understand engagement and utility of different behaviour change components.

Following iterative development work and user group testing and involvement, drawing on some online modules used in other LifeGuide interventions,^19^ we developed a bespoke intervention, called ‘e-coachER’ to support patients with chronic physical and mental health conditions who have been referred from primary care to an ERS to receive face-to-face support. Should the approach prove to be effective, there is considerable potential for the intervention to be scaled up for patients with obesity, hypertension, type 2 diabetes, osteoarthritis and risk of depression at probable low cost^25 26^ and also extend it for patients with other chronic medical conditions (eg, low back pain, heart disease, cancer).

## Aim and objectives

The overarching aim is to determine if e-coachER online support combined with usual ERS provides an effective and cost-effective approach to supporting increases in physical activity in people referred to ERS with a range of chronic conditions.

The specific objectives are as follows:

- To determine whether in the intervention arm compared with the control arm, there is an increase in the total weekly minutes of MVPA at 12 months postrandomisation.
- To determine whether in the intervention arm compared with the control arm there is an increase in the proportion of participants who:
  - take up the opportunity to attend an initial consultation with an exercise practitioner;
  - maintain objectively assessed physical activity from 4 to 12 months postrandomisation;
  - maintain self-reported physical activity from 4 to 12 months postrandomisation;
  - have improved health-related quality of life at 4 and 12 months postrandomisation.
- To quantify the additional costs of delivering the intervention and determine the differences in health utilisation and costs between the intervention and control arms at 12 months postrandomisation.
- To assess the cost-effectiveness of the intervention compared with control at 12 months postrandomisation (incremental cost per quality-adjusted life-year (QALY)) and over the lifetime perspective (incremental cost per QALY).
- To quantitatively and qualitatively explore whether the impact of the intervention is moderated by medical condition, age, gender and socioeconomic status, IT literacy or ERS characteristics.
- To quantitatively and qualitatively explore the mechanisms through which the intervention may impact on the outcomes, through rigorous mixed methods process evaluation and mediation analyses (if appropriate).

## Methods and analysis

This protocol is reported in accordance with the Standard Protocol Items: Recommendations for Interventional Trials guidance^27^ (http://www.spirit-statement.org/spirit-statement/) for protocols of clinical trials and TIDieR guidelines^28^ (http://www.equator-network.org/reporting-guidelines/tidier/) for intervention description.

### Study design and setting

**Figure 1:**
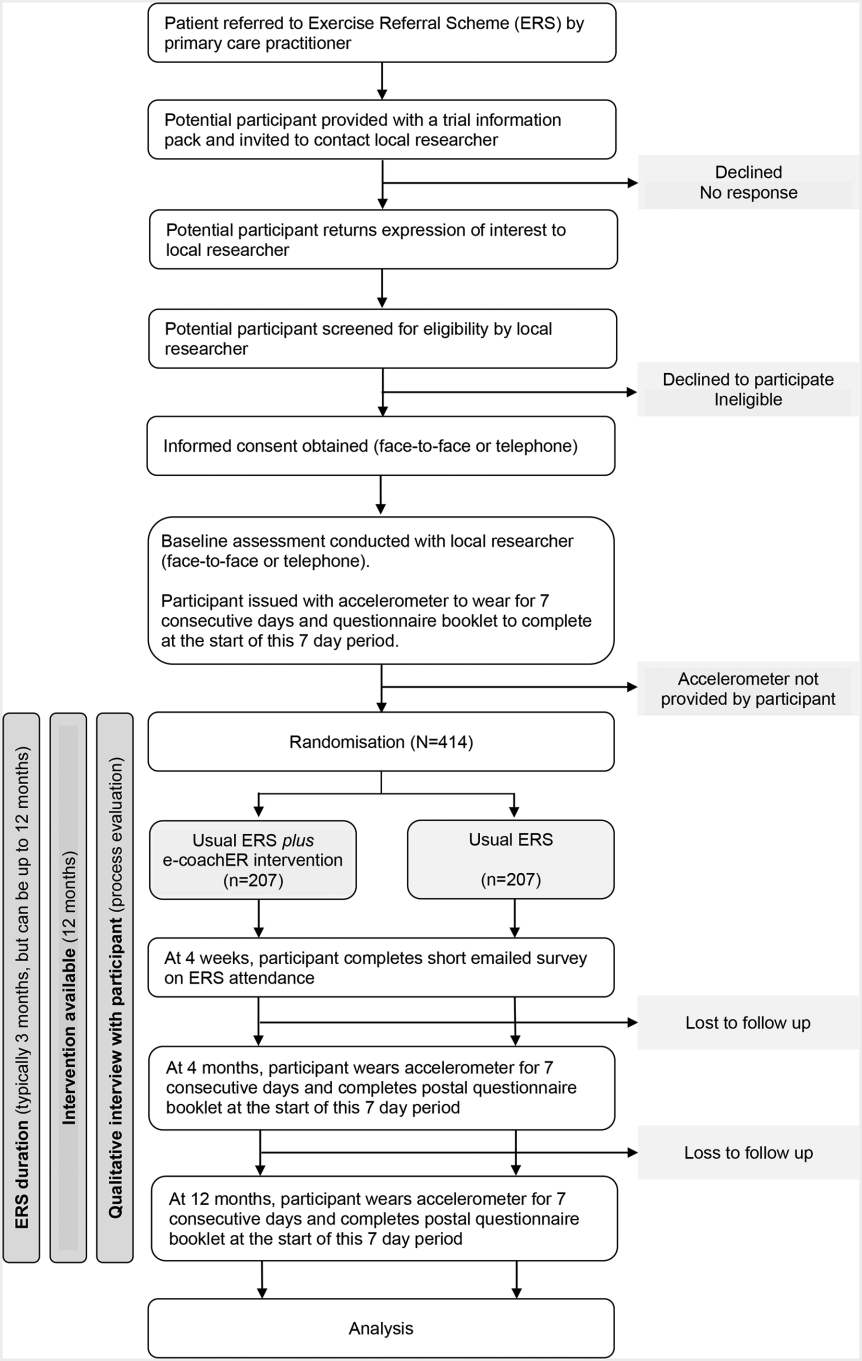
Trial design/participant pathway.

This is a multicentre parallel two-group randomised controlled trial with participant allocation to usual ERS alone (control) or usual ERS plus web-based behavioural support (intervention) with parallel economic and mixed methods process evaluations. The trial design is summarised in figure 1.

Recruitment to the trial will take place over a 21-month period (July 2015 to March 2017) in three areas in the UK, that is, Greater Glasgow, West Midlands and South West England (including Plymouth, Cornwall and Mid Devon). Only the latter includes some participants in more rural locations.

### Study population

The study population will include patients registered with a general practitioner (GP) surgery and who have been or are about to be referred to a local ERS for a programme of support to increase physical activity. Participants will be aged 16–74 years and have one of more of the following: obesity (body mass index (BMI), 30–40), a diagnosis of hypertension, prediabetes, type 2 diabetes, lower limb osteoarthritis or having a history of treatment for depression. Participants must also be categorised as ‘inactive’ or ‘moderately inactive’ based on the GP Physical Activity Questionnaire,^29^ be contactable via email, and have some experience of using the internet. Patients are excluded if they meet any of the following criteria: have an unstable, severe and enduring mental health problem or are being treated for an alcohol or drug addiction that may limit their involvement with the study, do not meet the eligibility criteria for their local ERS or are unable to use written materials in English unless a designated family member or friend can act as translator.

### Study procedures

#### Patient identification, approach and consent

Patients will be identified as potentially eligible for the trial (i) by healthcare professionals in primary care at the point of being actively referred to an ERS or having been opportunistically found to be eligible for an ERS at a consultation with the primary care practitioner, (ii) via a search of patient databases at the participating GP practices (conducted by the local Primary Care Research Network team), (iii) via patient self-referral to the GP arising from community-based publicity for the trial, (iv) by the ERS programme administrator on receipt of an ERS referral form from a GP practice or (v) by exercise advisors at the ERS service at enrolment on the ERS (with the patient’s consent, the exercise advisor will provide the local researcher with the patient’s contact details for the purposes of the trial).

Potentially eligible patients will be approached by the primary care practitioner or the local researcher, depending on how the patient was identified, or patients may self-refer to the local researcher in response to publicity campaigns. These various means of identification and approach are designed to accommodate the variation in usual care referral pathways to ERS across the participating sites and individual GP practices.

Amenable patients will be offered a study-specific Participant Information Sheet, either by post, via email or by hand (the route used will largely depend on the preference of the participating GP practice or ERS service). Interested patients will be asked to communicate their expression of interest to the local researcher via a prepaid reply slip, by telephone or by email. On receipt of an expression of interest, the local researcher will contact the potential participant by telephone to discuss the trial, confirm eligibility and take informed consent.

### Baseline assessment

Consented participants will attend a baseline assessment with the local researcher. This assessment will be conducted over the telephone, or in person at the GP practice or at the centre delivering the ERS or another convenient community location. Demographic data will be collected. The participant will be issued with a wrist-worn waterproof accelerometer (GENEActiv Original accelerometer http://www.geneactiv.org/) to wear constantly for one whole week (day and night), and a self-report questionnaire booklet to complete at the beginning of the week-long period. The accelerometer will be worn on the wrist of the non-dominant hand (ie, the hand not favoured for writing). After 1 week’s wear, participants will post the accelerometer and completed questionnaire to the Peninsula Clinical Trials Unit (CTU) in pre-addressed envelopes provided using a prepaid postal service. The measures collected at baseline and follow-up are shown in table 1.

**Table 1:** Schedule of baseline and follow-up measures

Measure	Baseline	Randomisation	4 weeks	4 months	12 months
Demographics	X				
Objectively measured physical activity (eg, minutes of MVPA in ≥10 min bouts, recorded by accelerometer)	X			X	X
Engagement with the ERS (uptake at 4 weeks, plus subsequent attendance at ERS, eg, number of sessions attended)			X X		
Engagement with e-coachER (captured from the LifeGuide platform)		X		X	X
Self-reported: MVPA (7-day recall of physical activity) Health and social care resource use Quality of life measures: 5-level Euroqol-5D (EQ-5D-5L), SF-12v2 Hospital Anxiety and Depression Scale	X			X	X
Process evaluation outcomes (eg, self-reported confidence to be physically active; perceived frequency and availability of support; perceived autonomy over choices; involvement in self-monitoring and planning physical activity)	X			X	X
Qualitative interviews as part of the process evaluation focusing on participants’ experiences with the ERS and the intervention (optional for participants)		————————————————X—————————————————			

ERS, exercise referral scheme; MVPA, moderate- to- vigorous physical activity.

### Randomisation

On receipt of the baseline accelerometer at the CTU after 1 week’s wear, participants will be randomised. Randomisation will be stratified by site with minimisation by the participant’s perceived reason for their referral to the ERS (ie, weight loss, diabetes control, reduce blood pressure, manage lower limb osteoarthritis symptoms, manage low mood/depression) and by self-reported IT literacy level on a visual analogue scale (ie, lower or higher confidence). To maintain allocation concealment, the minimisation procedure will retain a stochastic element and will be conducted using a secure, password protected web-based system.

### Blinding

The ERS practitioners should be unaware of trial participants’ treatment allocations. Blinding of participants is not possible, given the nature of the intervention. Given that the primary outcome is an objective measure of physical activity recorded by accelerometer, and the secondary outcomes will be assessed by participant self-completion questionnaire, the risk of assessor bias is likely to be negligible in this study. However, to minimise any potential bias, the statistical analysis will be kept blinded and the code for group allocation not broken until the primary and secondary analyses have been completed.

### Follow-up

At 4 weeks post-baseline, a short survey on initial uptake of the ERS will be administered via email.

At 4 and 12 months post-randomisation, participants will be sent an accelerometer and questionnaire booklet by post, along with a simple instruction sheet on how to wear the accelerometer, and a prepaid envelope to return the items to the CTU.

To maximise data completeness at follow-up assessments, participants will be sent standard letters/emails from the CTU: (i) 7 days before delivery of the accelerometer, (ii) 3 days into the 10-day recording window as a prompt for the participant to begin wearing the accelerometer (if not already doing so) and (iii) should the accelerometer not have been received at the CTU, at 3 and 5 weeks after issue as a reminder to post the accelerometer to the CTU. If the participant has not sent the accelerometer to the CTU after 6 weeks, the local researcher will telephone the participant to remind them to return the device. Participants who return the accelerometer to the CTU will receive an online/high street store voucher for £20 as a token ‘thank you’, to maximise response rates.

### Trial treatment/trial arms

#### Intervention: web-based support plus ERS (e-coachER)

**Figure 2:**
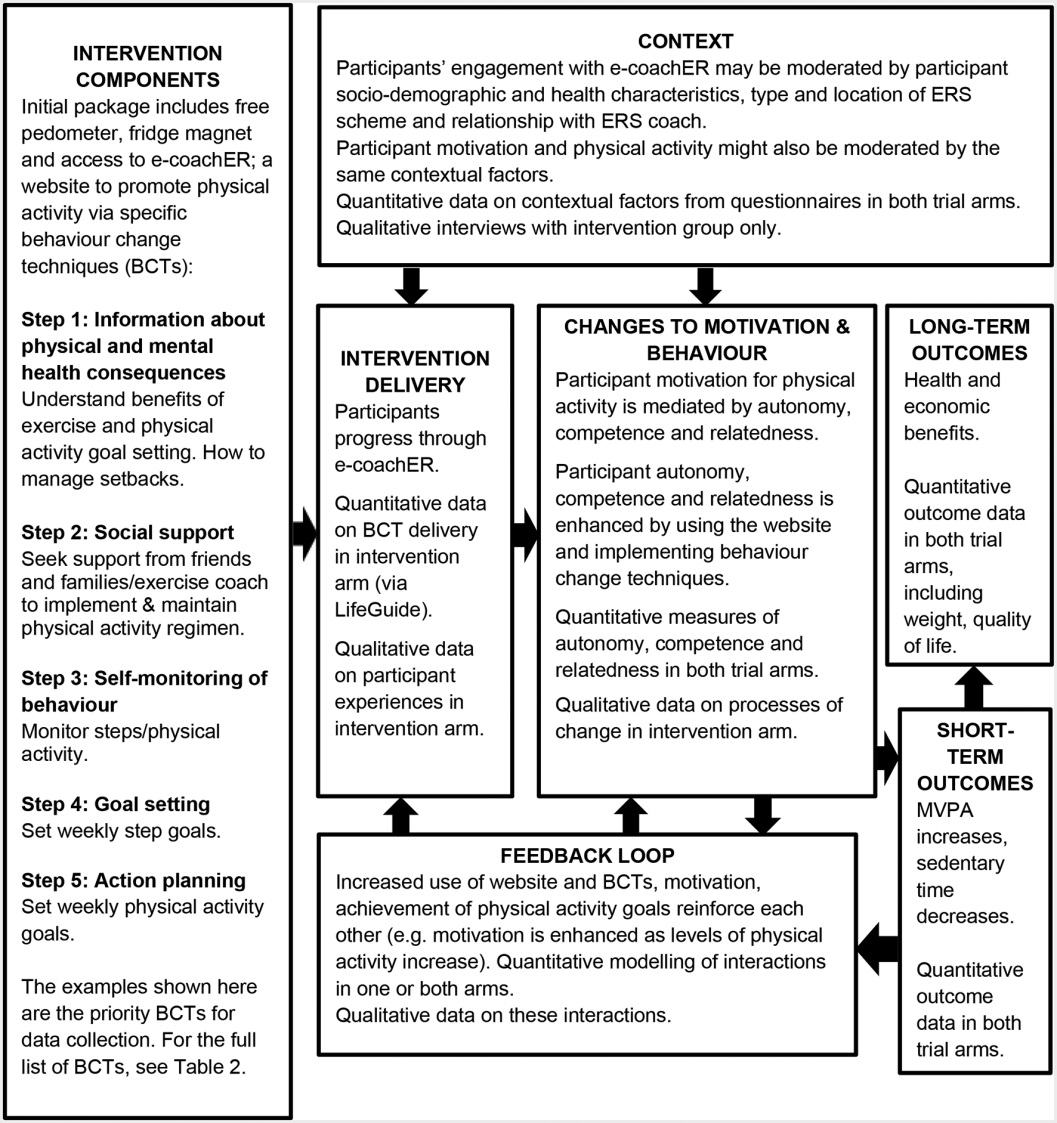
Logic model for e-coachER intervention. ERS, exercise referral scheme; MVPA, moderate- to- vigorous physical activity.

e-coachER is a web-based support package, which offers a range of interactive opportunities to enhance participants’ motivation to take up the ERS and to maintain a more physically active lifestyle, whether or not they engage with their local ERS. A logic model for the intervention is shown in figure 2.

e-coachER is primarily a self-delivered intervention and comprises the following components:

- A mailed ‘Welcome Pack’ that contains a user guide and the participant’s unique user log-in; a simple pedometer (step-counter) and a notepad to record daily physical activity (appended to a magnet with study-specific branding). Participants are encouraged to make use of the pedometer and the activity record sheets for self-monitoring and goal setting in conjunction with the e-coachER website.
- The e-coachER website (on the LifeGuide platform). At the core of e-coachER are seven ‘Steps to Health’ lasting approximately 5–10 min each, designed to: encourage participants to think about the benefits of physical activity (motivation); seek support from an ERS practitioner, friends/family and the internet (support/relatedness); set progressive goals; self-monitor physical activity with a pedometer and upload step counts or minutes of MVPA (self-regulation, building confidence/autonomy); find ways to increase physical activity more sustainably in the context of day-to-day life and deal with setbacks (building confidence). The sequential content, objectives and how this was implemented were mapped against a taxonomy for behaviour change techniques^30^ (table 2). Self-determination theory underpins the intervention with core aims in every step and interaction with participants, aiming to build confidence, autonomy and relatedness.^31^
  Participants are encouraged to use the e-coachER support package as an interactive tool by using preset or user-defined reminders to promote ongoing use of functions such as recording weekly physical activity (minutes of MVPA) and goal setting, and receive messages of encouragement. Prompts are sent to remind participants to review their goals. An absence of engagement (eg, failure to review a goal, or not signing into the website for 1, 2 and 4 weeks) triggers reminder emails to the participant.
  The website content will be locked prior to starting recruitment, with the exception of webpages displaying links to reputable generic websites for further information about the chronic conditions of interest and lifestyle, links to other websites and apps for self-monitoring health behaviour and health as well as modifiable listings of local opportunities to engage in physical activity.
  An avatar is used throughout the content to avoid having to represent a range of individual characteristics such as age, gender and ethnicity. The avatar delivers brief narratives to normalise and support behaviour change and encourage use of the e-coachER support package.
- To maximise accessibility and usage, a local researcher will provide technical support if requested. If a participant does not register on the e-coachER website within the first few weeks, the local researcher will contact the participant to offer support to register. If a participant requires technical support to resolve operational issues with the website (eg, requires a password to be reissued), participants will be referred to a centralised technician within the LifeGuide team.

**Table 2:** The e-coachER sequential process and objectives mapped against behaviour change techniques, and explanation of the implementation strategy

Sequential process	Performance objectives	Behaviour change techniques^30^	Implementation strategy
Welcome pack, pedometer and Introduction to web-based support for self-directed physical activity	To introduce the user to the philosophy of the website to become personal coach. Build on personal support provided by ERS using web-based platform. Support those who do not want to/cannot engage with ERS personnel. Support achievement of personal goals for physical activity to enhance health.	10. Self-monitoring	Explain philosophy of using website to become own personal coach. Links provided to local services and other self-help resources to highlight patient autonomy and choice. Offers e-coachER facilitator to help with using technology. Provide link to IT support from LifeGuide team.
Step 1: thinking about the benefits of physical activity	Elevate importance of physical activity.	82. Information about health consequences 83. Information about emotional consequences	Quiz to engage participants using positive framing. Provide evidence of multiple benefits of physical activity, especially for relevant health condition(s). Elicit and address concerns about physical activity, describing support given as part of ERS and by website.
Step 2: support to get active	To encourage user to access and create social support networks. To encourage user to take advantage of ERS and face-to-face support offered.	1. Social support (practical) 2. Social support (emotional) 3. Social support (unspecified)	Explain how to make the most out of the ERS support to learn how to become own personal trainer in future. Explain how user can create a personal ‘physical activity challenge’ and share it with family, friends, peers and exercise and health professionals. The patient may be encouraged to tell others about how e-coachER has been used to support behaviour change. Suggest ways of involving family or friends in long-term support for continued physical activity. Link to online sources of local support (eg, local walking or jogging group, or British Trust for Conservation Volunteers). How to use website to send personalised email/text reminders, motivational messages to self. Draw on positive normative beliefs; identify benefits of social interaction (companionship). Sharing personal physical activity challenge with others, involve friends and family, online local support links. Identify benefits of informational support (from ERS) in addition to emotional support from family and friends.
Step 3: counting your steps	To encourage and support the user to monitor step counts using a pedometer over a week. Emphasise personal experimentation.	10. Self-monitoring of behaviour	Provide guidance on how to count steps/use pedometer. Provide guidance on how steps can be implemented into lifestyle. Encourage self-monitoring using diary.
Step 4: making your step plans	To set explicit step count goals for the following week.	66. Goal setting (behaviour)	Give rationale and evidence for goal-setting for graded increase in physical activity. User sets specific, achievable goals for next week (eg, sessions completed, step count using the supplied pedometers). Links provided to local services and other resources.
Step 5: making your activity plans	To encourage and support the user to identify behavioural goals (types of activities).	68. Action planning	User selects walking or ‘other physical activities’ (which includes options for facility-based activity with practitioner support within ERS). Present options for facility and lifestyle-based activity. Sets specific, achievable goals for next week with a particular focus on avoiding days with less activity by planning walking or other activities. Keeping a physical activity diary.
Weekly goal and physical activity review	To promote adherence and graded increase in physical activity by providing tailored feedback and advice based on self-reported goal progress.	66. Goal setting behaviour 68. Action planning 69. Review behaviour goals	User records extent to which goals achieved in previous week, gets progress graph and personalised feedback. Praise for any goal achievement, encouragement to set a more challenging goal if not yet meeting target physical activity criteria. Encouragement where goals not attained, with links to webpages to assist with increasing motivation or confidence, selecting different activities or goals, making better plans, accessing support, overcoming setbacks (with links to relevant sessions below). Each session completed ends with new links to reputable information and resources (eg, NHS choices, condition-specific physical activity advice websites). Help user plan gradual increases in physical activity.
Step 6: finding ways to achieve your plans	To help the user harness their environment to provide support for physical activity. Identifying personal motivations, building confidence.	30. Restructuring the physical environment 31. Restructuring the social environment 32. Avoidance/reducing exposure to cues for behaviour	Make plan to use environment to automatically support physical activity (eg, fitness equipment in living room, route to work/shops that involves more physical activity, committing self to specific routine). Advise user on how to use website to send personalised email/text reminders, motivational messages. Overcoming barriers in work, leisure, home and travel. Building self-efficacy. Using smart phone apps for mobile support (eg, PowerTracker (c), MyFitnessPal (c)). Invite user to identify personal motivations for becoming more active.
Motivational messages (text and/or emails)	To provide reminders of user’s personal reasons (not necessarily health reasons) for becoming more active.	15. Prompts/cues	Invite user to write motivational message to be sent weekly or monthly detailing their own motivations for becoming more active.
Step 7: dealing with setbacks	To provide strategies for overcoming relapse in levels of physical activity.	5. Reduce negative emotions	Identify possible causes of relapse (eg, illness, holidays, change in work hours, new caring responsibilities) and plan ways to overcome barriers. Challenging catastrophic negative thoughts about lapses from intended physical activity. How to learn from a lapse and plan to avoid or overcome in future. Provide salient role models of people overcoming barriers to successfully engage with physical activity.

ERS, exercise referral scheme.

Intervention development, including piloting the Welcome Pack and developing an initial version of e-coachER, was built on wide ranging experiences from the development of other self-management interventions using the LifeGuide platform,^32^ and beta-testing over 7 months with input from service users. Co-applicants and researchers then provided feedback on a time-truncated version of the e-coachER website, and ERS patients provided feedback on a real-time version, for 5 months before the website was locked for the randomised controlled trial.

#### Usual care

There is currently no single model for ERS in the UK, but the predominant modes of delivery involve referral to a programme (eg, 10–12 weeks) of structured, supervised exercise at an exercise facility (eg, gym or leisure centre) or a counselling approach to support patients to engage in a variety of types of physical activity.^11^ ERS operate diversely to accommodate patient choice and local availability of facilities, the common goal being to reduce the risk of long-term metabolic, musculoskeletal and mental health conditions due to physical inactivity. The three participating sites were selected from different regions of the UK (different ERS providers) to provide diversity of approach; the schemes are described in table 3.

**Table 3:** Characteristics of the local ERS involved in the study

	South West England (predominantly Plymouth)	West Midlands (Birmingham)	Greater Glasgow and Clyde (GGC) Health Board Area
Population of city/locality and general characteristics	264 000 93% White British. Average age is 39 years. Plymouth has higher than average levels of poverty and deprivation (26.2% of population among the poorest 20.4% nationally). Life expectancy, at 78.3 years for men and 82.1 for women, is the lowest of any region in the South West of England.	1 244 438 White British (53.1%), Pakistani (13.5%) and Indian (6%). Birmingham is ranked the sixth most deprived local authority in the UK. Approximately 40% of the population lives in highly deprived areas. The average life expectancy in Birmingham is 77.1 years for males and 81.9 years for females.	1 161 370 GGC is the largest health board in the UK, comprising six local authority areas: 92.5% white, 5.3% Asian, Asian Scottish or Asian British, 1.2% African, 0.2% Caribbean or black, 0.4% mixed, 0.4% other. There is large variation in deprivation across GGC, but as a whole, it experiences higher than average levels of deprivation and poverty (34.4% population among the poorest 12.4% national average). Life expectancy at 74.9 years for males and 80.0 years for females, is the lowest in Scotland.
Number of centres/facilities where referrals are made to in the ERS	One main ERS run by Everyone Active in Plymouth and two smaller ones in rural locations. Referrals for ERS came from 31 local GP practices.	One main ERS, Be Active Plus run by Birmingham City Council Wellbeing Service. Referrals for ERS came from 286 local GP practices.	One main ERS (Live Active) delivered by six local leisure trusts in six local authority areas of GGC (Glasgow, East Renfrewshire, Renfrewshire, East Dunbartonshire, West Dunbartonshire and Inverclyde). Referrals are possible from any health professional in primary and secondary care.
Weeks, sessions and general details about ERS	Schemes vary from 6 to 12 weeks, attendees should commit to a minimum of two sessions/week in the gym with drop-in swimming, aquafit and gentle exercise group sessions available to all. All ERS referrals are risk assessed as low or medium risk. Those classed at medium risk may only attend a supervised session. Additionally, a ‘walking for health’ scheme is highlighted by one ERS provider.	Patients meet with a health and fitness advisor to discuss their preferences for physical activity and an individually tailored 12-week exercise programme is designed for them. Activities include the use of gyms, swimming, fitness classes, badminton and table tennis. The gyms are local authority or privately owned. Privately owned gyms are obliged to offer their facilities to Be Active Plus participants. Patients are also told about activities such as the use of parks and open spaces in Birmingham and walking to work, etc. Participants are also contacted after 3 and 6 months and a report is sent to their GP at their 12-week exit interview.	Patients meet with an ERS advisor for behavioural change support and to design a suitable physical activity plan. Patients are given information on a variety of physical activity options including those offered by leisure centres (eg, fitness classes, swimming, gym, etc) as well as health walks, home exercise, active travel, apps, etc and are able to offer specialist guidance on activities suitable for those with medical conditions and/or disabilities. Patients assessed as high risk at referral are screened by a cardiologist prior to being accepted to the scheme. There are fixed contact points of 1, 3, 6 and 12 months, but patients can choose how often they wish support (telephone, email or face-to-face) from the advisor in addition to these over a 12-month period.
Cost for patients in ERS (if applicable)	Costs vary related to age/concessions. 3 months ERS costs between £14.90 and £70 inclusive of all activities. Pay as you go: £2.10–£3.50 per session.	Patients are not charged for their assessment and support by the health and fitness advisor. The costs of the programme depend on chosen activities and leisure centre attended. Patients in receipt of state benefits or tax credits are eligible for a Passport to Leisure which entitles them to a 30% discount on most activities offered at Birmingham City Council run leisure centres, well-being centres and swimming pools. They can attend free Be Active sessions which take place at restricted times in leisure centres.	Live Active behavioural support is free to the patient for 12 months. If patients wish to use leisure facilities, they are entitled to access this at a concessionary rate (usually around 30% reduction).
Number of people referred to local ERS from 1 August 2015 to 31 March 2017 (ie, during the recruitment period of the study)	300	3470	6500
Most common primary reason for referrals (1 August 2015 to 31 March 2017)*	Depression/anxiety/stress: 24%	BMI>30: 28%	BMI≥30: 58%

*The data on primary reason for referral are subjective as many patients have multiple conditions and a practitioner may favour recording one condition (eg, obesity) rather than another (eg, low mood). Within the respective schemes, the quality of recording the referral reason also appears to be variable.

BMI, body mass index; ERS, exercise referral scheme; GP, general practitioner.

### Determination of sample size

In the absence of a published minimally important difference for MVPA, assuming a ‘small’ to ‘moderate’ standardised effect size of 0.35, we estimated that 413 participants are required at 88% power and a two-sided alpha of 5% assuming 20% attrition, or 90% power at a two-sided alpha of 5% allowing for 16% attrition (using ‘sampsi’ in STATA V.14). Given that the intervention is being delivered at the level of the individual participant, clustering has not been factored into the sample size calculation. Based on the baseline SD for MVPA total weekly minutes in ≥10 min bouts of 104–113,^33^ an effect size of 0.35 would correspond to a between-group difference of 36–39 min of MVPA per week.

### Measures

#### Primary outcome measure

The primary outcome is the number of weekly minutes of MVPA, in ≥10 min bouts, measured objectively by GENEActiv Original accelerometer,^34^ over 1 week at 12 months post-randomisation compared with the control group. To be included participants need to provide activity recorded over 4 days, including a weekend day, for at least 16 hours/day.

#### Additional measures

- Total weekly minutes of MVPA in ≥10 min bouts, measured objectively by accelerometer, over 1 week at 4 months.
- Achievement of at least 150 min of MVPA, measured objectively by accelerometer, over 1 week at 4 and 12 months.
- Achievement of at least 150 min of MVPA over 1 week using the self-reported 7-day Physical Activity Recall Questionnaire at 4 and 12 months.
- Self-reported weekly minutes of MVPA at 4 and 12 months.
- Average daily hours of sedentary behaviour measured objectively by accelerometer over 1 week at 4 and 12 months.
- Self-reported average daily hours of sleep over 1 week at 4 and 12 months.
- Self-reported health-related quality of life, assessed by the EQ-5D-5L^35^and SF-12v2^36^ at 4 and 12 months.
- Self-reported symptoms of anxiety and depression, assessed by the Hospital Anxiety and Depression Scale^37^ at 4 and 12 months.
- Uptake of the ERS by participant self-report at approximately 4 weeks and at 4 months, and from ERS records.
- Adherence to physical activity, using a composite measure to describe the proportion in each arm of the trial that achieved at least 150 min of MVPA in bouts of at least 10 min at 4 months and were still doing so at 12 months.

#### Self-reported survey process measures

- Single and multiple items, using Likert scales, to assess self-efficacy/confidence to be physically active, importance of being physically active, relatedness (perceived frequency and availability of support), perceived autonomy/control over physically active choices, involvement in self-monitoring and planning to do physical activity.
- In the intervention group, measures of engagement with e-coachER including whether or not the participant visits the website at least once, and whether they reach a stage of the online support to indicate they have set and reviewed at least one physical activity goal. Experience from engagement with other LifeGuide online interventions suggests there may not be an optimum dose of engagement.

#### Economic evaluation

- Cost-effectiveness. Incremental cost of the intervention to the NHS and incremental cost per change in minutes of MVPA (in ≥10 min bouts) and per QALY.
- An economic evaluation of e-coachER will be undertaken using NHS, personal social services, and patient perspective. The analysis will be twofold—short-term (within-trial) cost-effectiveness analysis (from baseline to 12 months postrandomisation) and long-term cost-effectiveness analysis (beyond-trial modelling of long-term expectations for cost-effectiveness), for e-coachER against ERS. The main outcome of the economic analysis will be an incremental cost per QALY (based on EQ-5D-5L). The short-term cost-effectiveness analysis will use resource use data for development of training of and input from a local LifeGuide facilitator, and central LifeGuide technician; provision and running of the exercise sessions at leisure centres and health and personal social service use. Data will be collected using the e-coachER monitoring system, key informant interviews (including trial manager), review of trial management records and participants’ questionnaires at baseline, 4 and 12 months. Unit costs will be taken from the NHS reference costs (eg, DH 2015/2016),^38^ standard unit costs^39^ and published literature. The long-term cost-effectiveness of e-coachER will be based on an existing policy-relevant decision analytical model.^40 41^ The analysis will account for the impact of physical activity on lifetime risk of developing coronary heart disease, stroke and type 2 diabetes.

### Process evaluation

The barriers to, and facilitators for, recruitment will be explored with participants in the early stages of the trial through qualitative interviews with local researchers at each site, and also via local researcher field notes of conversations with participants at various stages of the trial. Along with relevant supporting literature, this information will be used to optimise recruitment during the remainder of the trial.

Following guidelines for evaluating complex interventions,^42^ a nested mixed methods process evaluation will be undertaken, focussing on identifying factors relating to recruitment, engagement, acceptability, mechanisms and fidelity.

The assessment of barriers and facilitators in recruitment will involve the following:

1. Interviews with researchers about patient-reported reasons for joining the study or not;
2. Interviews with researchers about barriers to recruitment in the primary care setting, and among exercise referral practitioners.

The logic model shown in figure 2 will guide the process evaluation of the intervention. The logic model shows the types of data that will be collected, as well as the causal pathways proposed to contribute to behaviour change and intervention outcomes.

The assessment of intervention engagement and acceptability will involve the following:

1. Semi-structured interviews with up to 10% of the intervention group participants. A purposeful sampling framework will be used to ensure participants with a range of characteristics (gender, age, underlying health condition and trial centre) are invited to take part. Interviews will be conducted at different stages of participation in the trial, with each individual being invited to participate in telephone interviews and if appropriate follow-up interviews (up to a maximum of three telephone interviews over the course of the intervention period (approximately 4 months). Interviews will be recorded and transcribed and personal data or ways of identifying participants removed. Transcriptions will be imported into NVivo for data management purposes. The interview transcripts will be coded and thematic analysis performed to identify key findings. Analysis will initially focus on ‘top level’ themes, reflected in the intervention logic model. Analysis will follow the principles of Framework Analysis.^43^ Further in-depth analysis will also be undertaken in order to ensure emergent data, for example, from longitudinal cases, or condition-specific themes, are explored fully. The focus of the interview questions will be linked to the phase of the intervention, and seek to identify the perceived value of the ‘Welcome Pack’ and contents in helping to access e-coachER, the overall web-based support and each of the Steps to Health, in terms of functionality and utility to support behaviour change. Participants will be asked to identify if and how they thought e-coachER provided support for their ERS, and maintaining physical activity in addition to and beyond the ERS support. Ideas for additions or revisions to e-coachER will be requested. Questions will also focus on the participants’ perceived development of self-regulatory skills (eg, self-monitoring, goal setting) and the extent to which the intervention enhanced a sense of competence, autonomy and relatedness, thereby linking back to the aims and guiding principles of the e-coachER intervention.
2. The researchers will be asked to maintain field notes on any interactions with participants concerning engagement with the intervention, such as any difficulties faced with accessing the intervention website. Semi-structured interviews will be conducted by the qualitative researcher with the researchers at each recruitment site to identify participant barriers and facilitators to using e-coachER.
3. Engagement with the web-based e-coachER support system will be quantified. Metrics such as whether the participant registered, how far they progressed in the seven Steps to Health, visits to and time spent on different web pages and within each of the respective Steps, number of times step counts or amount of physical activity (eg, MVPA) were entered into e-coachER (ie, self-monitoring) and number of times goals were achieved and reviewed.
4. Changes in the process measures (see above) (eg, self-efficacy/confidence to be and importance of being physically active) from baseline to 4 and 12 months follow-up will be assessed and compared between intervention arms.
5. Mediation analysis to determine the extent to which changes in the process measures mediate the effect of the intervention on changes in physical activity at 4 and 12 months.

### Data handling

Data will be collected and stored in accordance with the Data Protection Act 1998/General Data Protection Regulation 2018.

#### Subject numbering

Following receipt of expression of interest, each patient will be allocated a unique number and will then be identified in all study-related documentation by their identification number and initials. A record of names, addresses, telephone numbers and email addresses linked to participants’ identification numbers will be stored securely on the study database for administrative purposes only.

#### Data collection

Data will be recorded on study-specific paper-based case report forms (CRFs) by the local researcher, and participants will complete a paper-based questionnaire booklet comprising validated and non-validated self-report outcome measures (listed in table 1).

Accelerometers will be configured for use prior to issue to participants by the local researcher at baseline and the CTU thereafter, using GENEActiv software. A recording window of 10 days, recording at 75 Hz, will be preset, thus accounting for transits in the post while optimising the battery life of the device.

Accelerometers received by the CTU following 1 weeks’ wear by the participant will be physically cleaned with liquid detergent (according to manufacturer’s instructions) before data are downloaded via GENEActiv software and linked to participant identification number. Accelerometers will then be issued to other participants in the trial as required.

Data on participants’ uptake of the ERS will be collected via a single use token-based authenticated email sent to participants at 4 weeks post-baseline. This will be a short survey requesting information on whether the participant has attended the initial consultation with the ERS advisor, and predefined reasons for non-attendance status, for example, appointment has been booked but not yet attended.

All persons authorised to collect and record study data at each site will be listed on the study site delegation logs, signed by the Principal Investigator.

#### Data entry

Original CRFs and questionnaire booklets will be posted to the CTU, with copies of the CRF retained at the study site. All data will be double-entered by CTU staff on to a password-protected SQL Server database and encrypted using Secure Sockets Layer. Double-entered data will be compared for discrepancies using a stored procedure and discrepant data will be verified using the original CRF. Incomplete, incoherent, unreadable or other problem data in the CRF pages will be queried by the CTU with study site staff during data entry to ensure a complete and valid dataset. Self-reported data in the questionnaire booklet will not be queried with participants.

The CTU may complete further validation of data items, perform logical data checks and raise further data queries after data collection has been completed. The final export of anonymous data will be transferred to statisticians for analysis after all data cleaning duties have been performed by the CTU.

### Data analysis plan

All analyses will be carried out using a detailed a priori statistical analysis plan. Analyses will be reported in full and in accordance with the Consolidated Standards of Reporting Trials (CONSORT) guidelines.^44^ Recruitment, uptake of the ERS, engagement with the intervention, outcome completion rates and study withdrawal will be reported (with 95% CIs). Baseline characteristics in the two trial arms will be reported.

The primary analysis will compare complete case outcomes between intervention and control arms groups according to the principle of intention to treat (ie, according to original randomised allocation) at 12 months adjusting for baseline outcome values and stratification and minimisation variables (recruitment site and disease indication).

Secondary analyses will be undertaken to compare groups at follow-up across all follow-up points (ie, 4 and 12 months) using a mixed effects repeated measures approach. In addition, we will seek to undertake secondary per-protocol analyses using a complier average causal effect approach to examine the impact of different levels of the adherence to the intervention.

Accelerometry data will be analysed with bespoke software to classify data into levels of physical activity intensity using accepted cut-points. Standard operating procedures will be applied to make a decision about dealing with missing data.

The primary analysis model will be extended to fit interaction terms to explore possible subgroup differences in intervention effect in stratification and minimisation variables and the predefined baseline characteristics. As not formally powered, these subgroup analyses will be regarded as exploratory and hypothesis-generating.

Sensitivity analysis, using multiple imputation and assuming unobserved measurements are missing at random will be conducted for both primary and secondary analyses to assess the likely impact of missing data on the primary and secondary outcomes at 12 months. Contemporary mediational analysis methods^45^ will be used to explore the impact of process outcomes identified in the planned intervention components, including engagement, use of behaviour change techniques and motivation and processes of change (eg, self-efficacy, autonomy, relatedness).

No interim analysis of primary or secondary outcomes is planned. No adjustment of p-values will be made to account for multiple testing, although the implications of multiple testing will be considered when evaluating the results of the analyses. Analysis of the primary outcome will be performed prior to all other analyses. All analyses will be undertaken using STATA V.14.2.

Checks will be undertaken to assess the robustness of models, including assessment of model residual normality and heteroscedasticity.

### Patient and public involvement

The research question was informed by patient and public involvement (PPI) over many years. Individual and group interviews were conducted with patients to identify the barriers and facilitators associated with ERS, and what additional support could help maintain physical activity for a variety of chronic conditions. Our extensive engagement with ERS practitioners allowed us to understand the individual variability and collective patient experience of ERS. This included one of the authors developing, delivering and adapting a training course for ERS practitioners based on their feedback.

The LifeGuide team worked extensively with PPI representatives to develop the appropriate support, concluding that ERS patients would appreciate additional support from an ERS to help them to further develop the independent motivation to maintain physical activity, involving a broad range of active options. Also, patients widely indicated that the LifeGuide web-based system can provide appropriate support for making health behaviour changes. Typically ERS can increase health inequalities by limiting access to those who have limited disposable income or have restricting physical and mental health conditions. The e-coachER system was designed to support those with such restrictions.

Patients were involved in the design of the study. A PPI group was involved in the initial development and refinement of the e-coachER web-based behavioural support. Patients with experience of being referred for an exercise programme, took part in focus groups and provided direct feedback on iterations of the e-coachER intervention during its development.

We engaged with over 20 ERS patients who volunteered to pilot the e-coachER Welcome Pack and provide feedback on the e-coachER website. A PPI representative was available to provide opinions on the study protocol and patient-facing documentation (eg, Participant Information Sheet) during the set-up of the study.

Patients are involved in the oversight of study progress and conduct via representation at periodic Project Management Group meetings and Trial Steering Committee meetings.

Results will be disseminated to study participants. At the end of the trial, a plain English summary of the study results will be made available to participants via a designated webpage on the Peninsula Clinical Trials Unit website, and emailed or posted to participants on request.

### Trial monitoring and oversight

A Project Management Group including the Chief Investigator, Principal Investigators, co-applicants, CTU Trial Manager, ERS advisor and PPI representative will meet quarterly to provide multidisciplinary input and oversight for the study.

A Trial Steering Committee (TSC) including an independent chair, independent clinicians and/or academics with relevant expertise, independent statistician/methodologist with relevant expertise and a representative contributing a patient/public perspective will oversee the conduct and scientific integrity of the trial. The TSC will review study progress and protocol adherence. Each committee will function in accordance with agreed terms of reference set out in a charter.

An independent Data Monitoring Committee (DMC) will monitor the safety and ethics of the trial by overseeing recruitment, primary outcome data completeness and serious adverse event data.

The committees will meet once before the start of the trial and approximately annually thereafter.

## Ethics and dissemination

### Safety considerations

The recording and reporting of non-serious adverse events in this study will not be required. Serious adverse events (SAE) will be captured via survey-specific items on hospital admissions in the questionnaire booklet at 4 and 12 months, that is, reason and duration of the inpatient stay, and self-reported relatedness of the SAE to participation in the trial; self-report independent of the questionnaire booklet; notification to the local researcher by the participant’s relative/advocate or notification by the participant’s GP.

Reports of SAEs will be provided to the CTU. The CTU will liaise with the local researcher who will be responsible for ascertaining further details about the SAE as appropriate. The Chief Investigator will report any SAE that is related (definitely, possibly or probably related) to the research procedures to the Research Ethics Committee within 15 days of becoming aware of the event. The CTU will prepare quarterly summaries of SAEs for review by the independent DMC and Sponsor.

### Dissemination plan

The findings of the study will be made publicly available through publication in relevant peer-reviewed journals and the NIHR Journals Library website; and presentation to the scientific community, patient support groups, the ERS services and NHS strategy forums at local and national level. The study is reported in accordance with CONSORT guidelines for publishing randomised trials and TIDieR guidelines for intervention reporting.

A plain English summary of the main study results will be made available for participants and other lay audiences.

### Changes to the protocol after the start of the trial

#### Primary outcome measure and sample size

The original protocol featured an internal pilot. During the internal pilot phase, 180 patients were to be recruited over 3 months to provide sufficient information to justify progression to a main trial. Progression from the internal pilot to the main trial was dependent on recruitment rate and engagement with the intervention according to the scenarios in table 4. In the main trial, an additional 1220 participants were to be recruited, giving a total of 1400 participants (recruited over 16 months).

**Table 4:** Internal pilot to main trial progression rules

Criteria	Scenario 3	Scenario 2	Scenario 1
% of internal pilot sample size target (180 patients) recruited	<65%	65%–79%	≥80%
Intervention engagement (% participants who access e-coachER at least once)	<65%	65%–79%	≥80%
Proposed action	No progression	Discuss with Trial Steering Committee and funder about progression and resources needed to achieve target.	Proceed to full trial.

The recruitment rate during the internal pilot phase was lower than expected, due to limitations on the time primary care practitioners had available to approach potential participants; delayed start at one of the research sites; poor uptake when patients were approached via a postal mailshot; high ineligibility rate among patients who were identified via a primary care database. In response to poor recruitment, the following strategies to increase recruitment were introduced:

- The inclusion criterion for BMI was aligned with the ERS entry (upper BMI limit for the trial was originally 35 and was raised to 40), and prediabetes was included as an inclusion criterion.
- Recruitment via the ERS service, which was already taking place at the site in Greater Glasgow, was adopted in the West Midlands and the South West in addition to recruitment via primary care.
- Incentive payments to participants (for returning an accelerometer) were increased from £10 to £20 per accelerometer.

Having implemented these measures, the conditions for progression in terms of recruitment rate and engagement with the intervention were not met by the end of the internal pilot phase, despite a 4-month extension period. A ‘recovery plan’ was developed in collaboration with the funders, based on amending the choice of primary outcome, and submitted in May 2016.

The original primary outcome was achievement of at least 150 min of MVPA measured objectively by accelerometer over 1 week at 12 months. This outcome was based on the findings of a systematic review of ERS^12 46^ demonstrating that trials had primarily reported their outcomes according to percentage of participants reaching the National Institute for Health and Care Excellence guidelines for physical activity level, that is, 150 min of MVPA per week. We estimated that recruiting 700 participants per group would allow us to detect a difference at 12 months follow-up of at least 10% (intervention group: 53% vs control group: 43%), assuming an attrition rate of 20% and small effect of clustering (intracluster correlation coefficient ICC: 0.006) at 90% power and 5% alpha. Thus, the original sample size was 1400 participants, to be recruited over 16 months.

From the outset, the TSC and DMC had recommended that this dichotomous primary outcome measure be replaced with a continuous variable; total weekly minutes of MVPA. This was because:

- A continuous primary outcome measure would be more relevant in this study population, in terms of detecting a small but clinically significant increase in minutes of MVPA.
- Based on sample size calculations, this would offer greater statistical power than to the categorical assessment of whether participants reach a threshold of 150 min of MVPA. This would therefore afford a reduction in sample size.

The TSC and funders agreed these changes (in August 2016) and the original sample size was reduced in accordance with this new primary outcome measure and revised sample size calculation, from 1400 to 413 participants (to be recruited over 21 months). A similar reduction in sample size has been incorporated into the qualitative component of the process evaluation work.

### Current study status

The e-coachER trial began recruiting patients in August 2015 and closed to recruitment in March 2017. Data collection is expected to be completed in March 2018 and results are expected to be published in September 2018.

## Supplementary Material

Reviewer comments

Author's manuscript

## References

[1] Public Health England. Physical inactivity: economic costs to NHS clinical commissioning groups London: Her Majesty’s Stationery Office, 2016.

[2] Department of Health. Start Active, Stay Active: A report on physical activity from the four home countries’ Chief Medical Officers. London: Department of Health, 2011.

[3] NICE. Obesity: Guidance on the prevention, identification, assessment and management of overweight and obesity in adults and children. London: National Clinical Guideline Centre, 2010.

[4] NICE. Hypertension: Clinical management of primary hypertension in adults. London: National Clinical Guideline Centre, 2011.

[5] NICE. Type 2 diabetes: The management of type 2 diabetes. London: National Clinical Guideline Centre, 2008.

[6] NICE. Osteoarthritis: The care and management of osteoarthritis in adults. London: National Clinical Guideline Centre, 2008.

[7] NICE. Depression: The treatment and management of depression in adults. London: National Clinical Guideline Centre, 2009.

[8] Bouchard C, Blair SN, Katzmarzyk PT, et al Less sitting, more physical activity, or higher fitness? Mayo Clin Proc 2015;90:1533–40. 10.1016/j.mayocp.2015.08.005 26422244

[9] Warburton DE, Bredin SS Reflections on physical activity and health: what should we recommend? Can J Cardiol 2016;32:495–504. 10.1016/j.cjca.2016.01.024 26995692

[10] Dunstan DW, Daly RM, Owen N, et al Home-based resistance training is not sufficient to maintain improved glycemic control following supervised training in older individuals with type 2 diabetes. Diabetes Care 2005;28:3–9. 10.2337/diacare.28.1.3 15616225

[11] British Heart Foundation National Centre for Physical Activity and Health. Section 2: A Snapshot of ER Schemes Operating in England, Scotland & Northern Ireland - 2006-2008: A Toolkit for the Design, Implementation & Evaluation of Exercise Referral Schemes: Loughborough University, 2010.

[12] Pavey TG, Taylor AH, Fox KR, et al Effect of exercise referral schemes in primary care on physical activity and improving health outcomes: systematic review and meta-analysis. BMJ 2011;343:d6462 10.1136/bmj.d6462 22058134 PMC3209555

[13] Pavey T, Taylor A, Hillsdon M, et al Levels and predictors of exercise referral scheme uptake and adherence: a systematic review. J Epidemiol Community Health 2012;66:737–44. 10.1136/jech-2011-200354 22493474

[14] Rouse PC, Ntoumanis N, Duda JL, et al In the beginning: role of autonomy support on the motivation, mental health and intentions of participants entering an exercise referral scheme. Psychol Health 2011;26:729–49. 10.1080/08870446.2010.492454 21827332

[15] Joseph RP, Durant NH, Benitez TJ, et al Internet-Based Physical Activity Interventions. Am J Lifestyle Med 2014;8:42–67. 10.1177/1559827613498059 25045343 PMC4103664

[16] Devi R, Singh SJ, Powell J, et al Internet-based interventions for the secondary prevention of coronary heart disease. Cochrane Database Syst Rev 2015;12:Cd009386 10.1002/14651858.CD009386.pub2 PMC10819100 26691216

[17] Davies CA, Spence JC, Vandelanotte C, et al Meta-analysis of internet-delivered interventions to increase physical activity levels. Int J Behav Nutr Phys Act 2012;9:52 10.1186/1479-5868-9-52 22546283 PMC3464872

[18] Morrison LG, Hargood C, Lin SX, et al Understanding usage of a hybrid website and smartphone app for weight management: a mixed-methods study. J Med Internet Res 2014;16:e201 10.2196/jmir.3579 25355131 PMC4259922

[19] Lloyd S, Dennison L, Morrison L, et al Losing weight online with POWeR: a randomised controlled trial of a web-based behavioural intervention in a community setting. The Lancet 2013;382:S62 10.1016/S0140-6736(13)62487-3

[20] Williams S, Yardley L, Wills GB A qualitative case study of LifeGuide: users' experiences of software for developing Internet-based behaviour change interventions. Health Informatics J 2013;19:61–75. 10.1177/1460458212458915 23486826

[21] Yardley L, Morrison LG, Andreou P, et al Understanding reactions to an internet-delivered health-care intervention: accommodating user preferences for information provision. BMC Med Inform Decis Mak 2010;10:52 10.1186/1472-6947-10-52 20849599 PMC2946266

[22] Little P, Stuart B, Hobbs FR, et al An internet-based intervention with brief nurse support to manage obesity in primary care (POWeR+): a pragmatic, parallel-group, randomised controlled trial. Lancet Diabetes Endocrinol 2016;4:821–8. 10.1016/S2213-8587(16)30099-7 27474214

[23] Greaves CJ, Sheppard KE, Abraham C, et al Systematic review of reviews of intervention components associated with increased effectiveness in dietary and physical activity interventions. BMC Public Health 2011;11:119 10.1186/1471-2458-11-119 21333011 PMC3048531

[24] Michie S, Abraham C, Whittington C, et al Effective techniques in healthy eating and physical activity interventions: a meta-regression. Health Psychol 2009;28:690–701. 10.1037/a0016136 19916637

[25] Anokye NK, Trueman P, Green C, et al The cost-effectiveness of exercise referral schemes. BMC Public Health 2011;11:954 10.1186/1471-2458-11-954 22200193 PMC3268756

[26] Benaissa M, Malik B, Kanakis A, et al Tele-healthcare for diabetes management: A low cost automatic approach: Conference proceedings: Annual International Conference of the IEEE Engineering in Medicine and Biology Society IEEE Engineering in Medicine and Biology Society Annual Conference, 2012:1290–3. 10.1109/EMBC.2012.6346174 23366135

[27] Chan AW, Tetzlaff JM, Altman DG, et al SPIRIT 2013 statement: defining standard protocol items for clinical trials. Ann Intern Med 2013;158:200–7. 10.7326/0003-4819-158-3-201302050-00583 23295957 PMC5114123

[28] Hoffmann TC, Glasziou PP, Boutron I, et al Better reporting of interventions: template for intervention description and replication (TIDieR) checklist and guide. BMJ 2014;348:g1687 10.1136/bmj.g1687 24609605

[29] Ahmad S, Harris T, Limb E, et al Evaluation of reliability and validity of the General Practice Physical Activity Questionnaire (GPPAQ) in 60-74 year old primary care patients. BMC Fam Pract 2015;16:113 10.1186/s12875-015-0324-8 26329981 PMC4557746

[30] Michie S, Richardson M, Johnston M, et al The behavior change technique taxonomy (v1) of 93 hierarchically clustered techniques: building an international consensus for the reporting of behavior change interventions. Ann Behav Med 2013;46:81–95. 10.1007/s12160-013-9486-6 23512568

[31] Deci EL, Ryan RM Handbook of self-determination research. Rochester, New York: University of Rochester Press, 2002.

[32] Yardley L, Morrison L, Bradbury K, et al The person-based approach to intervention development: application to digital health-related behavior change interventions. J Med Internet Res 2015;17:e30 10.2196/jmir.4055 25639757 PMC4327440

[33] Harris T, Kerry SM, Victor CR, et al A primary care nurse-delivered walking intervention in older adults: PACE (pedometer accelerometer consultation evaluation)-Lift cluster randomised controlled trial. PLoS Med 2015;12:e1001783 10.1371/journal.pmed.1001783 25689364 PMC4331517

[34] Powell C, Carson BP, Dowd KP, et al Simultaneous validation of five activity monitors for use in adult populations. Scand J Med Sci Sports 2017;27:1881–92. 10.1111/sms.12813 27905148

[35] Herdman M, Gudex C, Lloyd A, et al Development and preliminary testing of the new five-level version of EQ-5D (EQ-5D-5L). Qual Life Res 2011;20:1727–36. 10.1007/s11136-011-9903-x 21479777 PMC3220807

[36] Ware JE, Kosinski M, Turner-Bowker DM, et al How to score version 2 of the SF-12 health survey (with a supplement documenting version 1). Lincoln, RI; Boston, Mass: QualityMetric Inc; Health Assessment Lab, 2002.

[37] Zigmond AS, Snaith RP The hospital anxiety and depression scale. Acta Psychiatr Scand 1983;67:361–70. 10.1111/j.1600-0447.1983.tb09716.x 6880820

[38] Department of Health. NHS Reference Costs 2014 to 2015, 2015.

[39] Curtis LA Unit costs for health and social care University of Kent. Canterbury: Personal Social Services Research Unit, 2014.

[40] Anokye NK, Lord J, Fox-Rushby J Is brief advice in primary care a cost-effective way to promote physical activity? Br J Sports Med 2014;48:202–6. 10.1136/bjsports-2013-092897 24352807 PMC3913207

[41] Campbell F, Holmes M, Everson-Hock E, et al A systematic review and economic evaluation of exercise referral schemes in primary care: a short report. Health Technol Assess 2015;19:1–110. 10.3310/hta19600 PMC4781341 26222987

[42] Moore GF, Audrey S, Barker M, et al Process evaluation of complex interventions: Medical Research Council guidance. London: MRC Population Health Science Research Network, 2014. 10.1136/bmj.h1258 PMC4366184 25791983

[43] Ritchie J, Spencer L Qualitative data analysis for applied policy research Analysing Qualitative Data. London: Routledge, 1994.

[44] Schulz KF, Altman DG, Moher D CONSORT 2010 statement: updated guidelines for reporting parallel group randomised trials. BMJ 2010;340:c332 10.1136/bmj.c332 20332509 PMC2844940

[45] Emsley R, Dunn G, White IR Mediation and moderation of treatment effects in randomised controlled trials of complex interventions. Stat Methods Med Res 2010;19:237–70. 10.1177/0962280209105014 19608601

[46] Pavey TG, Anokye N, Taylor AH, et al The clinical effectiveness and cost-effectiveness of exercise referral schemes: a systematic review and economic evaluation. Health Technol Assess 2011;15:1–254. 10.3310/hta15440 PMC4781450 22182828

