# Supplementary material for: Multicentred randomised controlled trial of an augmented exercise referral scheme using web-based behavioural support in individuals with metabolic, musculoskeletal and mental health conditions: protocol for the e-coachER trial
Source: BMJ Open. 2018 Sep 21;8(9):e022382. doi: 10.1136/bmjopen-2018-022382 (PMC6157530; doi:10.1136/bmjopen-2018-022382)
Supplement: Reviewer comments [file bmjopen-2018-022382.reviewer_comments.pdf]

PEER REVIEW HISTORY

BMJ Open publishes all reviews undertaken for accepted manuscripts. Reviewers are asked to complete a checklist review form (**http://bmjopen.bmj.com/site/about/resources/checklist.pdf**) and are provided with free text boxes to elaborate on their assessment. These free text comments are reproduced below.

ARTICLE DETAILS

**TITLE (PROVISIONAL)**	A multi-centred randomised controlled trial of an augmented exercise referral scheme using web-based behavioural support in individuals with metabolic, musculo-skeletal and mental health conditions: Protocol for the e-coachER trial.
**AUTHORS**	Ingram, Wendy; Webb, Douglas; Taylor, Rod; Anokye, Nana; Yardley, Lucy; Jolly, Kate; Mutrie, Nanette; Campbell, John; Dean, Sarah; Greaves, Colin; Steele, Mary; Lambert, Jeffrey; McAdam, Chloe; Jane, Ben; King, Jennie; Jones, Ray; Little, Paul; Woolf, Anthony; Erwin, Jo; Charles, Nigel; Terry, Rohini; Taylor, Adrian

This article was not externally reviewed at BMJ Open. Protocols that have been independently assessed prior to submission to BMJ Open are usually fast-tracked to publication on the grounds that further substantial changes will not be possible. This independent assessment will usually be external, independent review for both a substantial grant award from a non-commercial or government funder and ethics approval.
